# Silencing Viral Infection

**DOI:** 10.1371/journal.pmed.0030242

**Published:** 2006-07-25

**Authors:** Derek M Dykxhoorn, Judy Lieberman

## Abstract

The authors describe recent progress and obstacles to harnessing RNA interference to prevent or treat viral infection.

## The Need for New Antiviral Therapies

Viruses are formidable targets for drug development. As obligate intracellular parasites that take over the host cellular machinery to further their own agenda, they seem to understand the intimate workings of cellular pathways better than those seeking to develop treatments against them. It is difficult to inactivate a virus without doing harm to the host cell.

Only in the past decade with the concerted efforts to control the HIV/ AIDS epidemic have antiviral drugs come into their own. By targeting viral proteins and pathways unique to the viral life cycle, it has become possible to interfere with viral infection and replication for a few viruses without unacceptable host cell toxicity. However, antiviral drugs have only been developed for a handful of viruses, and none of these antiviral drugs is completely effective. Viral resistance, sequence diversity, and drug toxicity are significant problems for all antiviral therapies. In this article, we discuss recent progress and obstacles to harnessing RNA interference to prevent or treat viral infection.

HIV-1 and hepatitis B and C virus infections are responsible for significant global epidemics. Moreover, there is no treatment for most acute viral infections, including ones that cause hemorrhagic fever, encephalitis, and death. The prospect of global pandemics caused by newly emerging infections, such as SARS coronavirus or avian influenza, in the setting of economic development and changing ecology, has made the rapid development of novel antiviral therapies an international priority. It is not surprising, then, that as soon as RNA interference (RNAi) was discovered to work in mammalian cells, researchers honed in on attempts to harness this ancient antiviral mechanism to prevent or treat viral infection.

## How RNAi Works

RNAi uses small double-stranded RNAs to silence genes bearing a complementary sequence. Although endogenous gene silencing operates through multiple mechanisms, including mRNA cleavage, inhibition of translation, and epigenetic modifications of chromatin, mRNA cleavage is the most efficient mechanism and is the mechanism being harnessed for antiviral therapies.

**Figure 1:**
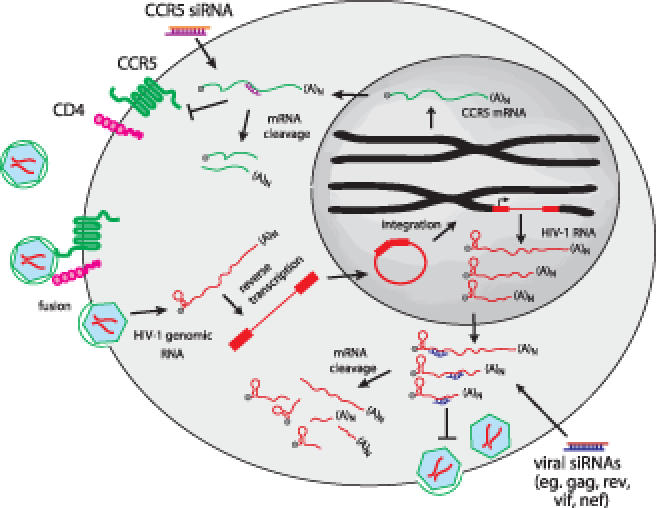
Possible Targets for Suppressing HIV Replication siRNAs can be used to silence either viral mRNAs or host genes required for viral entry or replication. This figure shows some possible targets for suppressing HIV replication. Silencing *CCR5* can prevent viral entry, while any viral gene can also be silenced to interfere with viral production. siRNAs could also target the viral long term repeat or even the proviral genomic RNA prior to integration, although whether this can be done is controversial. Targeting multiple genes will enhance viral suppression and reduce the chances of viral escape by sequence mutation.

Small RNAs, either exogenous small interfering RNAs (siRNAs) or endogenous microRNAs, are taken up by a cytoplasmic RNA-induced silencing complex (RISC), which cleaves one strand, leaving the remaining unpaired guide strand to search for mRNAs bearing complementary sequences. Once recognized, if the target site on the mRNA has nearly perfect complementarity to the guide siRNA, the mRNA is cut by an Argonaute endonuclease in the RISC and then degraded, silencing the expression of the protein it encodes. Typically protein expression is reduced but not completely eliminated. The RNAi machinery is present in all cells, where it is used to regulate the expression of key genes involved in cell development, differentiation, and survival. Small RNAs can be readily designed to target any gene, whether an endogenous host gene or foreign viral gene. For example, for HIV virtually all of the nine HIV gene products have been shown to be capable of being silenced, and siRNAs targeting the viral long terminal repeat are also effective ( Figure 1).

## RNAi Is an Ancient Antiviral Defense

In organisms that lack an adaptive immune response to pathogens, RNAi is an important defense against viral infection. In plants and flatworms, the genes encoding key RNAi pathway endonucleases Dicer and Argonaute have developed into gene families. These families may have evolved to provide molecules dedicated to controlling viral infection and preventing chromosomal insertion of rogue genetic elements, such as transposons, to supplement the activities of other homologous RNAi molecules required for regulating endogenous gene expression. Over 90% of plant viruses use double-stranded RNA at some point in their life cycle, making them especially vulnerable to Dicer cleavage and suppression by RNAi. Plant viruses have developed mechanisms to bypass RNAi, most prominently by synthesizing viral proteins that bind and sequester siRNAs; other mechanisms have been described and additional ones are still being uncovered.

Although the importance of RNAi in antiviral defense has been clearly shown in plants, worms, and flies, whether or not mammals use RNAi to defend against viruses is still uncertain, but likely. Several mRNAs encoded by a primate retrovirus contain complementary sequences that are targeted by an endogenous microRNA. Moreover viral variants with a mutated target sequence replicate more efficiently. Several examples of mammalian virus strategies for interfering with RNAi have been described, suggesting the viruses are trying to bypass an antiviral host response [ 1]. One strategy, analogous to the suppression of silencing exhibited by plant viruses, is elaboration of proteins, such as NS1 of influenza and E3L of vaccinia, that bind to and inactivate double-stranded RNAs. Another strategy, employed by adenovirus, is copious expression of a microRNA that binds to and inactivates the microRNA export and Dicer processing machinery.

Mammalian viruses also take advantage of RNAi to carry out their replication strategies. In particular, several herpesviruses encode microRNAs that seem to be involved in regulating viral latency. Viruses may use microRNAs to regulate sequential patterns of gene expression much like other organisms use microRNAs to regulate key genes that determine progression from one developmental state to another. *SV40*-encoded microRNAs direct the cleavage of early *SV40* transcripts, which encode T antigens [ 2]. Hepatitis C virus makes use of the microRNA *miR-122*, which is efficiently expressed in liver cells and matches a sequence in the viral 5′ untranslated region, to promote its replication [ 3]. Since viruses have evolved to exploit intrinsic host molecular pathways to promote their own replication, they will be an important tool for probing how microRNAs regulate gene expression even in uninfected cells. Interfering with either virally encoded or host microRNAs required for efficient viral replication may provide a novel strategy for antiviral therapeutics. A recent study shows the feasibility of inhibiting microRNA function in vivo using cholesterol-conjugated 2′-O-methyl siRNAs [ 4].

## Guiding Principles for Choosing siRNAs to Suppress Viruses

RNAi can be used to suppress viral replication by targeting either viral genes or host genes needed for viral replication. Silencing viral genes such as viral polymerases or master regulators of viral gene transcription (which are essential for fundamental viral processes), or silencing viral genes that act early in the viral life cycle, may more effectively suppress viral replication than targeting late genes or accessory viral genes that contribute to pathogenesis (but that are not vital for viral replication). In vitro studies have shown the usefulness of RNAi in vitro to suppress virtually every class of virus, whether a virus uses double-stranded, single-positive, or negative-strand RNA or DNA for its genome. Algorithms for siRNA or short hairpin RNA (shRNA) design provide guidance for designing effective sequences, but this process is imperfect (with a success rate of over 60%). The algorithms do not predict which sequences will be most effective at silencing their target gene. Nor do they predict which sequences maximize viral inhibition and minimize sequence-specific off-target effects arising from unintended silencing of partially homologous genes or from sequence-specific binding to Toll-like receptors, which triggers an inflammatory response. The best candidate siRNA sequences need to be determined by experiment. Off-target silencing of genes with partial homology is impossible to avoid because inhibition of translation may involve complementarity to just a seven-nucleotide stretch of the siRNA. However, translational inhibition generally has only a small impact on gene expression, can be minimized by avoiding sequences that target important genes, and is unlikely to be an important impediment to the clinical use of RNAi, although clinically significant toxicity from off-target effects will be difficult to predict in advance. Off-target effects caused by incorporation of the passenger strand, instead of the antisense strand, of the siRNA into RISC can be avoided by designing the siRNA ends to favor RISC uptake by the intended active strand.

## Addressing the Obstacles of Viral Sequence Diversity and Escape Mutation

Important obstacles to using RNAi as the active principle for antiviral treatments are the related issues of viral sequence diversity and potential viral escape from gene silencing by sequence mutation. RNA viruses, such as poliovirus or HIV, may be particularly susceptible to escape mutation because of the low fidelity with which their genomes are replicated [ 5, 6]. These problems can be circumvented by silencing host genes required for viral entry or replication.

However, targeting host genes may lead to unacceptable host cell toxicity, unless RNAi can be selectively induced only in infected cells or transiently in vulnerable subsets of cells. This might be achieved by targeting siRNAs exclusively into vulnerable cells or by regulating expression of shRNAs by using an inducible promoter that is activated by a viral gene product. A recent strategy that uses an antibody fragment fusion protein to bind and deliver siRNAs only into cells bearing the cell surface receptor recognized by the antibody can deliver siRNAs and silence gene expression specifically in HIV-infected cells [ 7]. Expression of an shRNA designed to silence a host protein required for HIV replication might be induced only upon HIV infection by using a TAT-dependent promoter [ 8]. For HIV infection, the viral coreceptor *CCR5* might be an exceptional host gene that can be silenced without toxicity, since individuals homozygous for a genetic mutation that functionally inactivates *CCR5* are completely asymptomatic.

Since the morbidity and mortality associated with many viral infections may be secondary to stereotypic overly exuberant inflammatory or immune responses, silencing host genes proximal in these pathways provides another possible type of intervention. For example, hepatitis B and C are not cytopathic viruses, but trigger hepatitis when activated immune cells expressing FasL infiltrate the liver, where the infection upregulates the death receptor Fas on hepatocytes, making them prime targets for immune-mediated apoptosis. Silencing Fas in hepatocytes or FasL in immune cells might provide an effective immune-modulating therapy to circumvent chronic liver cell damage [ 9].

When viral genes are targeted, the related problems of viral sequence diversity and potential escape mutation can be circumvented by choosing highly conserved sequences whose mutation might result in impaired viral fitness. This may be possible even for a highly diverse and mutation-prone virus, like HIV, in which many short sequences are conserved even at the nucleotide level. By targeting a highly conserved HIV *vif* sequence, researchers were able to suppress a variety of primary viral isolates chosen at random to represent five distinct viral clades [ 10]. In a recent study by Kumar and colleagues published in *PLoS Medicine*, a sequence conserved among a variety of flaviviruses silenced both Japanese encephalitis virus and West Nile virus [ 11].

Another approach for preventing viral escape mutation is to combine siRNAs targeting multiple genes, much as effective drug therapy for HIV requires combining antiviral drugs targeting different steps in HIV replication. For the encephalitis viruses, Kumar and colleagues' study suggests it might be possible to administer a cocktail of siRNAs that could effectively suppress a variety of likely suspect viruses before a definitive diagnosis is made, thus avoiding a dangerous delay in treatment. For any particular virus, a cocktail of siRNAs targeting viral and host genes whose products act at different points in the viral life cycle is likely to act synergistically to suppress viral replication more effectively and hence reduce the chance of viral escape mutation [ 12]. However, the cellular RNAi machinery has limited capacity since silencing can be suppressed by an excess of irrelevant double-stranded small RNAs, a strategy used by adenovirus for suppressing RNAi [ 13]. Similarly, gene therapy vectors that express high amounts of shRNAs can cause toxicity by interfering with the nuclear export of endogenous microRNAs [ 14]. Therefore, there is probably a limit to the number of siRNAs that can be effectively incorporated into any treatment. However, no published study has yet defined this limit.

## In Vivo Delivery

The key obstacle to harnessing RNAi as a treatment is getting small RNAs into the cytoplasm of cells in vivo. This can be accomplished either by figuring out a way to introduce small RNAs into cells as small molecule drugs (siRNA) or by using a plasmid or viral vector encoding an shRNA so that it will be processed like the endogenous microRNA precursors. The small molecule approach is best suited to short-term interventions, while gene therapy can potentially be used for long-term silencing via expression from an integrated transgene. To treat chronic infections, such as HIV or hepatitis C, a gene therapy strategy may make sense. However, concerns about safety and controlling gene expression make gene therapy less practical for immediate applications.

The key obstacle to harnessing RNAi as a treatment is getting small RNAs into the cytoplasm of cells in vivo.

**Figure 2:**
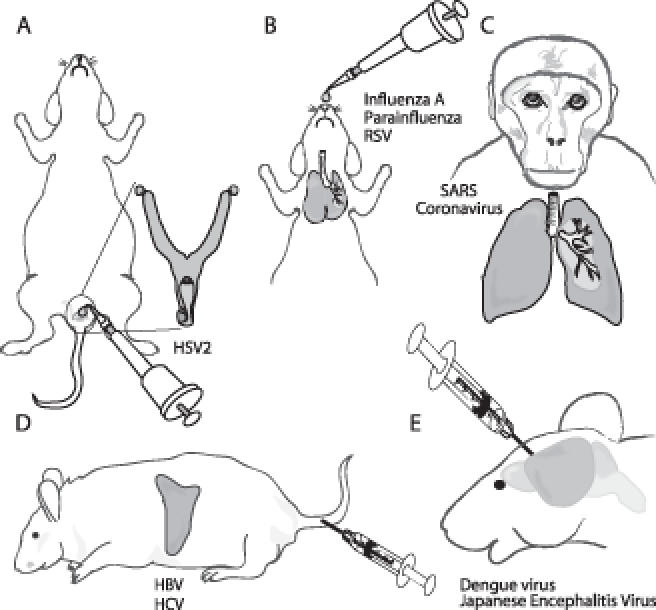
siRNA Antiviral Therapy in Animal Models (A) Intravaginal application of siRNAs mixed with a transfection lipid inhibits sexual transmission of HSV-2 in mice [ 17]. (B) Intranasal or intratracheal administration of siRNAs, either by themselves or mixed with lipids, protects against respiratory infection in mice [ 12, 13, 15]. (C) Intranasal administration of siRNAs in rhesus macaques protects against SARS coronavirus infection [ 16]. (D) Intravenous injection of siRNAs incorporated into specialized liposomes inhibits a hepatitis B virus replicon [ 19]. (E) Intracerebral injection of siRNAs protects against flavivirus encephalitis [ 10]. HSV-2, herpes simplex virus type 2; HBV, hepatitis B virus; HCV, hepatitis C virus; RSV, respiratory syncytial virus.

Although small RNAs are not taken up by themselves into most cells in the body, the mucosal tissues of the body are an important exception. siRNAs mixed with a cationic lipid or even by themselves are efficiently taken up by epithelial cells in the lung and vagina ( Figure 2). In the genital tract, siRNAs are taken up deep into the lamina propria and the underlying stroma. The mechanism of uptake is not understood. Most viral infections are transmitted through mucosal surfaces, which provides the opportunity to intervene to prevent or treat viral infections during transmission or acute infection. In mice, local instillation of siRNAs targeting viral genes has shown striking protection against respiratory challenge with influenza, parainfluenza, and respiratory syncytial viruses [ 15–17]. The potential for siRNAs to treat viral infection has also been shown in primates challenged with the SARS coronavirus [ 18]. These encouraging animal experiments have led to the recent first phase I study of siRNAs designed to treat viral infection. This study will begin to test the potential of RNAi for treatment of acute respiratory syncytial virus infection, a leading cause of death and serious morbidity in newborn children.

Another mucosal tissue in which siRNAs have been shown to prevent viral infection is the female genital tract, where siRNAs targeting HSV-2 genes protected mice from lethal HSV-2 infection [ 19]. siRNA penetrated deep into the cervicovaginal tissue, and silencing persisted for over nine days. Mice were even protected when siRNA administration was delayed until 3 hours after viral challenge. This study suggests the possibility that a persistent antiviral state might be achieved in the genital tract to prevent sexual transmission, reactivation, or even oncogenesis for a variety of sexually transmitted viruses, such as HSV-2, HIV, and human papillomavirus. Topical application of antiviral siRNAs might also be effective at other localized and accessible sites of viral infection, such as the upper respiratory tract, eye (i.e., herpes keratitis), or skin (i.e., warts).

Although therapeutic delivery of siRNAs and silencing is clearly feasible at mucosal surfaces, systemic treatment of viruses that are disseminated or infect tissues deep within the body is more difficult because of the challenge of efficiently transducing cells in vivo. The main impediments to systemic uptake are the difficulty in traversing the cell membrane and the short half-life of intravenously injected siRNAs. Several potential solutions to the former problem have been described recently, including covalent conjugation of the passenger siRNA strand to a cell-targeting moiety, noncovalent association of siRNAs with cell-targeting molecules, and incorporation of siRNAs into liposomes or other nanoparticles [ 7, 20, 21]. The short half-life, which is caused by both rapid renal filtration and endogenous ribonuclease digestion, can be readily addressed by incorporating siRNAs into complexes or particles and by minor chemical modifications of the RNA backbone. Early studies showing inhibition of hepatitis viral replication required introducing siRNAs via a high-volume bolus (termed hydrodynamic injection) that causes right-sided heart failure and is not suitable for clinical use. Although siRNAs can be effectively introduced into an organ, such as the liver or kidney, by selective catheterization of the principal vein draining the tissue [ 22], this is invasive and costly. Similarly, the intracerebral injection approach used to deliver siRNAs targeting flaviviruses is not likely to be used clinically. Intravenous low-pressure injection of chemically modified siRNAs incorporated into modified liposomes reduced replication of a hepatitis B virus replicon in mice [ 21]. This study was the first demonstration of a systemic antiviral effect using a method of injection that is clinically feasible.

It is likely that some of the other approaches developed for systemic siRNA delivery described above will also work for silencing viral infection. However, so far, achieving a noninvasive method to introduce siRNAs into the brain, as would be needed for treating viral encephalitis, remains an elusive goal.

**Five Key Papers on Harnessing RNAi for Antiviral Therapy**

- Novina CD, Murray MF, Dykxhoorn DM, Beresford PJ, Riess J, et al. (2002) siRNA-directed inhibition of HIV-1 infection. Nat Med 8: 681–686. One of the initial papers to use RNAi in vitro to inhibit HIV; the paper introduced the concept of inhibiting both viral and host genes to suppress viral entry and replication.
- Gitlin L, Karelsky S, Andino R (2002) Short interfering RNA confers intracellular antiviral immunity in human cells. Nature 418: 430–434. An early paper that showed in vitro inhibition of poliovirus and pointed out the potential problem of viral escape.
- Bitko V, Musiyenko A, Shulyayeva O, Barik S (2005) Inhibition of respiratory viruses by nasally administered siRNA. Nat Med 11: 50–55. A study in mice that showed efficient silencing and in vivo effectiveness of nasally administered uncomplexed siRNAs for the clinically important respiratory syncytial virus.
- Li BJ, Tang Q, Cheng D, Qin C, Xie FY, et al. (2005) Using siRNA in prophylactic and therapeutic regimens against SARS coronavirus in Rhesus macaque. Nat Med 11: 944–951. The first study to show therapeutic efficacy of siRNAs in a nonhuman primate to treat respiratory infection by the SARS coronavirus.
- Palliser D, Chowdhury D, Wang QY, Lee SJ, Bronson RT, et al. (2006) An siRNA-based microbicide protects mice from lethal herpes simplex virus 2 infection. Nature 439: 89–94. A study that shows the potential for vaginal application of siRNAs to prevent or treat sexually transmitted infections.

## Abbreviations

RISC: RNA-induced silencing complex
RNAi: RNA interference
shRNA: short hairpin RNA
siRNA: small interfering RNA
